# Sentinel Node Biopsy Using Two Concurrent Labeling Techniques (Radioactive Tracer With/Without Blue Dye vs. Indocyanin Green-ICG) in Early-Stage Endometrial Cancer Patients (TESLA–1): A Prospective Observational Study CEEGOG EX-02

**DOI:** 10.3390/cancers17101606

**Published:** 2025-05-09

**Authors:** Maja Pakiz, David Cibula, Dariusz Grzegorz Wydra, Jaroslav Klat, Michal Zikan, Olga Matylevich, Renata Poncova, Anna Abacjew-Chmylko, Andrej Cokan, Martina Romanova, Filip Frühauf, Sambor Sawicki, Leyla Al Mahdawi, Roman Kocian, Zuzanna Mascianica, Jure Knez, Lukas Dostalek, Paulina Zygowska, Jiri Slama, Marek Murawski, Daniela Fischerova, Radoslaw Owczuk, Andraz Dovnik

**Affiliations:** 1 Department for Gynecologic and Breast Oncology, University Medical Center Maribor, 2000 Maribor, Slovenia; maja.pakiz@gmail.com (M.P.);; 2 Department of Obstetrics, Gynecology and Neonatology First Faculty of Medicine, Charles University and General University Hospital in Prague, 18000 Prague, Czech Republic; 3 Department of Gynecology, Obstetrics and Neonatology, Medical University of Gdansk, 80-204 Gdansk, Poland; 4 Department of Obstetrics and Gynecology, Gynecological Oncology and Endocrinological Gynecology, University Clinical Centre in Gdansk, 80-952 Gdansk, Poland; 5 Department of Obstetrics and Gynecology, University Hospital Ostrava and Faculty of Medicine University of Ostrava, 70800 Ostrava, Czech Republic; 6 Department of Gynecology and Obstetrics, Charles University—First Faculty of Medicine and University Hospital Bulovka, 18000 Prague, Czech Republic; 7 Gynecologic Oncology Division, NN Alexandrov National Cancer Centre of Belarus, 223040 Minsk, Belarus; 8 Clinical Department of Gynecologic Surgery and Oncology, Wroclaw Medical University, 50-367 Wroclaw, Poland; 9 Department of Anaesthesiology and Intensive Therapy, Medical University of Gdansk, 80-210 Gdansk, Poland

**Keywords:** sentinel lymph node, endometrial cancer, tracer

## Abstract

Background: While sentinel lymph node (SLN) biopsy has been integrated into international guidelines for endometrial cancer, a standardized technique is still lacking. This study addresses whether the concurrent use of two tracers, technetium-99 (Tc) and indocyanine green (ICG), administered intracervically through distinct techniques, enhances the performance of SLN biopsies. As the blue dye is used routinely by some centers, it can be used alone; however, our analysis focused on only Tc and ICG (as is used in the majority of centers). Methods: A prospective multicentric observational study was designed to evaluate the unilateral detection rate, bilateral detection rates, sensitivity, and consistency of SLNs when using both tracers simultaneously in patients with early-stage endometrial cancer. Results: Our findings demonstrated that the simultaneous use of ICG and Tc significantly outperformed the use of either tracer alone. Unilateral detection rates were 69.2% for Tc, 84.9% for ICG, and 88.4% for both. Bilateral detection rates were 57.0% for Tc, 77.9% for ICG, and 83.6% for both. Additionally, the incidence of “empty pockets” was low with both tracers, at 2.7%. Notably, the concurrent application of both tracers identified instances where the Tc-labeled sentinel node differed from the ICG-labeled sentinel node. Conclusions: The combined use of Tc and ICG in SLN biopsy for early-stage endometrial cancer significantly enhances detection rates and reduces the occurrence of “empty pockets”, potentially decreasing the need for site-specific lymphadenectomy.

## Simple Summary

Sentinel lymph node (SLN) biopsy in endometrial cancer patients has largely replaced systematic pelvic lymphadenectomy as it is associated with lower rates of intraoperative morbidity. The optimal protocol for SLN biopsy is still debatable and varies among various institutions. Our study demonstrated that the concurrent use of ICG and Tc is associated with higher unilateral and bilateral detection rates compared to either one of these methods. We found very low rates of SNL detection discrepancy between the two tracers. The use of two tracers might additionally decrease the need for site-specific lymphadenectomy.

## 1. Introduction

Since 2003, sentinel lymph node (SLN) biopsy has progressively supplanted systematic lymphadenectomy as the standard method for surgical staging in endometrial cancer [1]. According to the most recent versions of the ESGO-ESTRO-ESP and National Comprehensive Cancer Network (NCCN) guidelines, SLN biopsy is now the preferred approach for lymph node evaluation in patients with low to intermediate risk factors and may also be employed in high-risk disease [2,3].

When SLN biopsies for endometrial cancer were first introduced, the radioactive tracer technetium-99 (Tc) and/or blue dye were the primary agents available. Indocyanine green (ICG) became available at a later stage. Various studies have reported on the use of different tracers, multiple tracer administration routes (including cervical, fundal, myometrial, and peritumoral via hysteroscopy), and varying algorithms for lymph node staging. Up until 2020, when our study was being planned, only 1 of the 27 studies published on SLN biopsies had utilized a combination of Tc and ICG, although it did so without addressing the consistency or inconsistency between the tracers in mapping SLNs [4,5]. Among the major prospective trials published to date, three (FIRES, SHREC, and SENTOR) exclusively used ICG [6,7,8] while only one trial (SENTI-ENDO) explored the use of a radioactive tracer alone [9]; however, none of these trials employed both tracers simultaneously. A 2017 systematic review concluded that the performance of ICG supports recommendations that it be used as the preferred tracer in these biopsies, although the exclusive use of ICG is not universally endorsed [10]. While both the ESGO-ESTRO-ESP and NCCN guidelines recommend the cervical route for tracer administration and suggest that ICG should be the preferred detection method, the specific details surrounding the optimal protocol for SLN biopsies remain incompletely defined.

Consequently, unresolved scientific questions persist, largely due to the lack of standardization in SLN biopsy methodology and the critically low quality of recent meta-analyses assessing the utility of SLN biopsies for staging endometrial cancer [11]. Notably, there is a paucity of trials evaluating the performance and potential benefits of using both technetium-99 (Tc) and indocyanine green (ICG) simultaneously.

The objective of our study was to determine whether the concurrent use of two tracers (Tc and ICG), administered intracervically using two distinct techniques, enhances the performance of SLN biopsies. Specifically, we aimed to assess improvements in SLN detection rates, mapping accuracy, sensitivity, and the consistency of SLN identification with each tracer.

## 2. Materials and Methods

This study was designed to be a prospective multicentric observational academic study conducted within CEEGOG centers. The study was registered at Clinicaltrial.gov (NCTO4665544). All national/institutional ethic committees approved this study according to their local legislation. The patients were enrolled at the time of surgery planning and signed informed consent forms.

### 2.1. Inclusion and Exclusion Criteria

The inclusion criteria were defined as (a) histologically proven endometrial cancer (any tumor type), (b) apparent early-stage endometrial cancer with intermediate- or high-risk prognostic factors (deep myometrial invasion or G2/G3 disease or non-endometrioid histological type) and no evidence of bulky or suspicious pelvic/para-aortic lymph nodes or distant metastases on preoperative conventional imaging studies, (c) a performance status ECOG of 0–1, (d) age ≥18 and ≤85, (e) a history of second primary cancer only if it had been >5 years with no evidence of disease, and (f) approved and signed informed consent form. The exclusion criteria were pregnancy, a desire for fertility sparing, and a history of pelvic or abdominal radiotherapy.

### 2.2. The Tracers’ Application

Radioactive tracer (Tc) was applied at least 2 and at most 16 h before surgery using four syringes each containing 1 mCi (40 MBq), which was injected submucosally into the four quadrants of the cervix at the 12, 3, 6, and 9 o’clock positions (the cumulative dose of radiotracer was 4 mCi (150 MBq)). If the blue dye was added, 2 mL of non-diluted blue dye was applied slowly and submucosally into the cervical stroma in the four quadrants, in identical positions to those of the radiotracer, at the beginning of the surgery. As some centers routinely use blue dye with Tc we allowed the use of blue dye; however, our final analysis and the protocol of this study focused only on Tc and ICG together (as is the preferred method in the majority of centers). At the start of the surgery, 4 mL of ICG (4 mg/mL) was injected into the cervix at 3 and 9 o’clock, 2 mL at each site. Then, 1 mL was injected into the cervical stroma at a depth of 1–2 cm and another 1 mL was injected into the subepithelial layer of the cervical stroma.

### 2.3. Surgical Protocol

When performing the surgery, radioactive/hot tissue was mapped before turning on the infrared light. After all radioactive/hot tissue was located, the infrared light was turned on and the fluorescent tissue was detected. The tissue potentially containing SLN was marked as (a) hot or blue or both; (b) fluorescent; or (c) hot or blue AND fluorescent. Each tissue sample was sent for pathological examination. A systematic pelvic and/or paraaortic lymphadenectomy was performed in defined regions (external iliac, obturator, common iliac, presacral, paraaortic inframesenteric, and paraaortic infrarenal) and all tissue samples from each individual region were sent for separate pathological examinations.

### 2.4. Endpoints of This Study

The primary endpoint of this study was the side-specific detection rate, also referred to as the unilateral detection rate. Secondary endpoints included the sensitivity of sentinel lymph node (SLN) biopsy for pelvic lymph node staging, the number of detected SLNs, the anatomical localization of the detected SLNs, and the bilateral detection rate. The unilateral detection rate for both technetium-99 (Tc) and indocyanine green (ICG) was defined as the proportion (percentage) of hemipelvises in which radioactive (hot) and/or fluorescent tissue was identified, respectively. The true unilateral detection rate was defined as the number of pathologically confirmed instances of lymph nodes within the removed hot or fluorescent tissue per hemipelvis, with “empty pockets” referring to tissues designated as SLNs that did not contain any lymph nodes upon pathological examination. The incidence of “empty pockets” was calculated as the percentage of tissue samples identified as “SLNs” that did not contain lymph nodes, revealing only fat upon histopathological examination.

The sensitivity of SLN biopsy for lymph node staging was determined by defining false negatives as cases where the SLN biopsy was negative despite at least one of the other pelvic and/or paraaortic lymph nodes being positive. The bilateral detection rate was defined as the percentage of patients in whom SLNs were detected in both hemipelvices or in one hemipelvis along with the paraaortic region. The agreement between the Tc and ICG mapping of SLNs was analyzed. All SLNs were subjected to ultrastaging according to the following protocol: tumor foci larger than 2 mm were classified as macrometastases, foci between 0.2 and 2 mm were classified as micrometastases, and foci smaller than 0.2 mm were classified as isolated tumor cells (ITCs).

### 2.5. Selection of Sites

To ensure the quality of the centers included in this study, their general requirements were defined as follows: ≥20 patients with invasive endometrial cancer suitable for the TESLA–1 trial must be treated in the center per year; equipment must be available for laparoscopic or open-surgery radioactive labeling and ICG labeling; and each surgeon participating in the study must have experience with at least 30 total hysterectomies with bilateral salpingo-oophorectomy and pelvic/para-aortic lymphadenectomy and experience with at least 15 patients with endometrial or cervical cancer, where the SLN was successfully mapped using all of the tracers and pathological SLN ultrastaging available.

### 2.6. Statistical Analysis

We analyzed general data (age, number of pregnancies, menopausal status, histopathological tumor characteristics), issues with the tracers’ applications, the surgical approach, conversion rate, and presence of intrauterine injuries and compared the tracers’ performance in relation to obesity (body mass index). This study was powered to a minimum required sample size of N = 165 hemipelvices (83 patients); we selected from the analyzed scenarios those with a 5% difference between sensitivities and a minimum proportion of discordant pairs (5.1%) due to the expectation of a high correlation between the methods.

Statistical analysis was performed using Python 3.9, with Pandas library utilized for data manipulation and SciPy library for statistical tests. Additionally, 95% confidence intervals (95% CI) for proportion estimates were calculated using the Clopper–Pearson exact method. The equality of the sensitivities of diagnostic tests were compared using a Z-test for two populations’ proportion estimates. To compare the proportion estimates between the two groups, Fisher’s Exact Test was employed, while for multiple groups the Fisher–Freeman–Halton test was used. Statistical significance was determined at a threshold of *p* < 0.05.

## 3. Results

**Figure 1:**
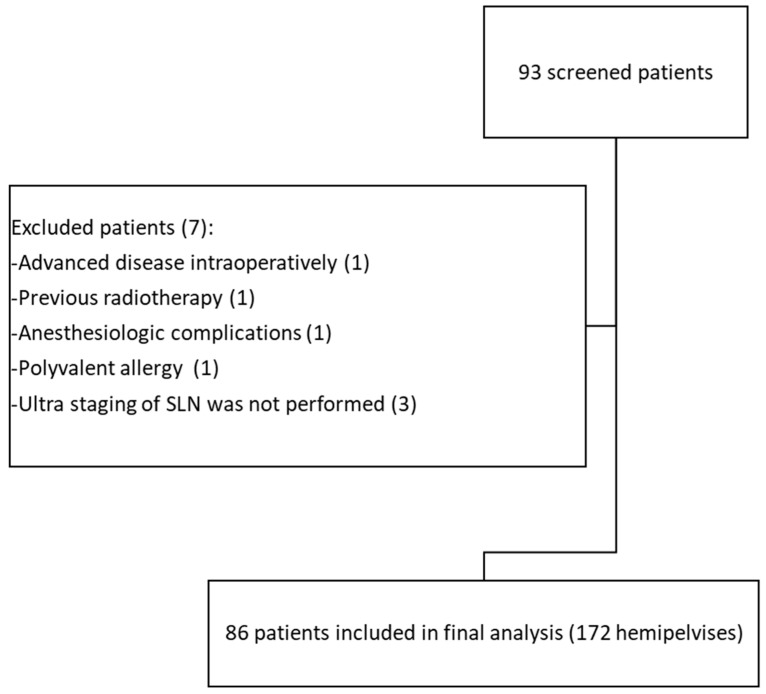
Flowchart of this study’s patient selection process.

A total of 93 patients were screened, of whom 86 were included in this study (24 from Prague, 24 from Gdansk, 20 from Maribor, 9 from Bulovka, 8 from Ostrava, and 1 from Minsk). A flowchart of the patient selection process, along with the patients’ general data, histopathological characteristics, and disease stage, are shown in Figure 1 and Table 1.

A laparoscopy was performed in 66 of 86 patients (75.6%), with conversion to laparotomy occurring in 7 cases. Intraoperative complications occurred in 6 of 86 patients (6.7%). Among these, one patient experienced a Grade I respiratory adverse event, one experienced a Grade II cardiac event, three experienced Grade III hemorrhagic events, and one experienced a Grade IV hemorrhagic event. Paraaortic lymph node dissection (LND) was performed in 41 of 86 patients (47.7%).

The application of both tracers was completed without complications in all 86 patients.

The average number of sentinel lymph nodes (SLNs) removed per tissue sample was 2.83, with a mean of 3.03 SLNs removed per patient. On the right side of the pelvis, SLNs were identified in the external iliac region in 46.5% of cases, in the obturator region in 39.5%, and in the common iliac region in 12.8%. Similarly, on the left side, SLNs were detected in the external iliac region in 53.5% of cases, in the obturator region in 40.7%, and in the common iliac region in 11.6%. SLNs were detected below the inframesenteric artery in the paraaortic region in 2.3% of patients and in the presacral region in 4.7%; no SLNs were detected between the inframesenteric and renal arteries.

Positive lymph nodes were found in 18 patients (20.9%), including 5 patients who had both positive SLNs and non-SLNs, 11 patients with only positive SLNs, and 2 patients with negative SLNs and positive non-SLNs. Among the 261 lymph nodes removed and analyzed, only 28 were positive (15 with macrometastasis, 5 with micrometastasis, and 8 with isolated tumor cells [ITCs]). When focusing exclusively on SLNs, 17 were positive (7 with macrometastasis, 3 with micrometastasis, and 7 with ITCs).

“Empty pockets” were identified in 1 of 7 tissue samples (14.3%) examined using technetium-99 (Tc) alone (95% CI: 0.4–57.9%), in 4 of 37 tissue samples (10.8%) examined using indocyanine green (ICG) alone (95% CI: 3.0–25.4%), and in 3 of 111 tissue samples (2.7%) examined using both Tc and ICG (95% CI: 0.6–7.7%). The true unilateral detection rate was 69.2% for Tc (95% CI: 61.71–75.99%), 84.9% for ICG (95% CI: 78.64–89.88%), and 88.4% when both tracers were utilized (95% CI: 82.61–92.75%), which is a statistically significant difference (*p* < 0.001).

The bilateral detection rate was 56.98% for Tc (95% CI: 45.85–67.61%), 77.91% for ICG (95% CI: 67.67–86.14%), and 82.56% when both tracers were employed (95% CI: 72.87–89.90%), which is also a statistically significant difference (*p* < 0.001).

The sensitivity of SLN biopsies in accurately reflecting nodal status was 98.26% (95% CI: 93.86–99.79%) with Tc, 98.26% (95% CI: 93.86–99.79%) with ICG, and 98.26% (95% CI: 93.86–99.79%) when both tracers were combined. No statistically significant differences were observed among these sensitivities.

Table 2 displays the performance outcomes of the tracers as stratified by body mass index (BMI), with patients categorized into two groups: BMI < 30 and BMI ≥ 30.

Among the three patients with positive paraaortic lymph nodes, one patient had negative sentinel lymph nodes (SLNs) and a positive lymph node in the paraaortic infrarenal region (skip paraaortic lymph node metastasis) while one other patient had an isolated positive SLN in the paraaortic region.

In 5 out of 172 hemipelvises (3%), the SLN identified by technetium-99 (Tc) differed from that identified by indocyanine green (ICG). Specifically, in one patient, one SLN was located in the left external iliac region, while the second SLN was found in the left obturator region. In the other four patients, one SLN was detected in the right external iliac region and another SLN was found in the right obturator region. All SLNs and lymph nodes assessed following lymphadenectomy (LND) were negative in these cases.

## 4. Discussion

### 4.1. Summary of Main Results

The findings of our study have demonstrated the superiority of the simultaneous use of indocyanine green (ICG) and technetium-99 (Tc) compared to the use of either tracer alone. The combination of tracers yielded improved outcomes in terms of unilateral detection rates (69.2% for Tc, 84.9% for ICG, and 88.4% for both tracers) and bilateral detection rates (57.0% for Tc, 77.9% for ICG, and 83.6% for both tracers) and resulted in the lowest incidence of “empty pockets”. Moreover, the concurrent application of both tracers revealed instances in which the Tc-labeled sentinel node differed from the ICG-labeled sentinel node.

Our results confirmed that the use of a single tracer is associated with a non-negligible incidence of “empty pockets”, with incidence rates of 14% for samples identified solely by radioactivity and 10% for samples identified exclusively by fluorescence. In contrast, when both tracers indicated the same tissue sample, the probability of lymph nodes being missed decreased to just 2.7%. This difference was particularly pronounced among patients with a body mass index (BMI) ≥ 30, where the incidence of “empty pockets” was 14% for samples in which only radioactivity was used, 21% for those examined using only fluorescence, and merely 1.7% when both tracers were used on the same tissue sample.

### 4.2. Results in the Context of Published Research

For sentinel lymph node (SLN) biopsy to effectively replace systematic lymphadenectomy, the detection of SLNs is paramount. The unilateral and bilateral detection rates observed in our study are consistent with those reported in the literature [5,10,12]. However, a novel and significant aspect of our study is the emphasis on “empty pockets,” which refers to instances where radioactive and/or fluorescent tissue is detected, but histopathological examination reveals an absence of lymph nodes. If this occurrence is sufficiently frequent, it may significantly impact the overall detection rate of this method.

Published data typically do not specify the frequency of this particular phenomenon. Given that obesity is a known risk factor for endometrial cancer and that adipose tissue can impair visualization, it aligns with the existing literature that a BMI ≥ 30 is associated with a higher failure rate in SLN detection, with “empty pockets” being present in 8% of obese patients compared to 4% in patients with a BMI < 30 [13]. Recently, a hybrid tracer has been developed that combines both a radioactive component and indocyanine green (ICG). In a published study focusing on endometrial cancer, the radioactive component successfully detected SLNs in 97.1% of patients, while the fluorescent component identified SLNs in 80% of cases, with no instances of “empty pockets” reported [14]. Therefore, one potential benefit of utilizing both tracers simultaneously is the improved detection of SLNs in the general population and especially in obese patients.

Our results reaffirmed the superiority of using both tracers simultaneously in terms of the bilateral detection rate, which was nearly as high as the published unilateral detection rates and surpassed the outcomes from trials utilizing hybrid tracers [14]. The bilateral detection rate is crucial for evaluating the sensitivity of SLN biopsy in patients with endometrial cancer, given that the uterus is a midline organ with bilateral lymphatic drainage; this is also why a site-specific lymphadenectomy is recommended when SLNs are not mapped [2,3,15,16]. The high bilateral detection rate achieved by using both tracers may therefore further reduce the necessity for site-specific lymphadenectomy.

Our findings demonstrated the high sensitivity of the SLN method, which consistently ranged from 98.3% to 98.6%, regardless of the tracer used. This sensitivity remained robust in obese patients (97%) and was 100% in patients with a BMI of less than 30. Our data exhibited slightly better results than those previously published, aligning with the complete SLN algorithms reported in the SHREC and SENTOR trials [7,8].

What our results demonstrate, and what has not been specifically published to date, is the discrepancy between SLNs labeled with Tc and ICG, which occurs in 3% of hemipelvices.

One proposed model of uterine drainage suggests the existence of two pathways: the upper paracervical pathway (UPP), which drains into medial external and/or obturator lymph nodes, and the lower paracervical pathway (LPP), which drains into internal iliac and/or presacral lymph nodes [17]. According to this anatomical framework, it has been proposed that the bilateral detection of at least one SLN from both the UPP and LPP should be a target [17].

### 4.3. Strengths and Limitations

The major strengths of our study include its prospective protocol, which uniquely emphasized the detection of radioactive sentinel lymph nodes (SLNs) prior to activating the infrared camera. This study was multicentric, reflecting real-life clinical scenarios. Quality-related criteria were established in advance. However, as the sample size was determined based on the unilateral detection rate, the number of patients included in the study is relatively small. In this era of precision medicine, and when considering the potential clinical significance of all lymph node metastases, there is a clear need for further research to address the existing lack of harmonization in performing SLN biopsies in endometrial cancer patients. Another limitation is that both modalities (Tc and ICG detection) are not available in all centers; the need to carry out further research is thus associated with the need to include centers overseen by different research groups across the world.

### 4.4. Implications for Practice and Future Research

We hypothesize that the use of two tracers with different half-lives, administered via distinct intracervical techniques, may enhance the likelihood of detecting all SLNs [14]. In our study, all lymph nodes involved in this discrepancy were negative, indicating that the lack of concordance between the SLNs did not impact the overall sensitivity of the biopsy. However, despite it being a rare occurrence, this discrepancy leads to a risk of false negative SLN results being generated when utilizing a single tracer.

The ultrastaging of sentinel lymph nodes (SLNs) represents a primary advantage of the SLN biopsy method, as ultrastaging has been shown to upgrade the disease stage in at least 5% of patients [18] and was responsible for identifying 37% of all patients with nodal involvement in a meta-analysis conducted by Burg et al. [19]. Our findings indicated that isolated tumor cells (ITCs) were more prevalent than micrometastases. While previous studies have suggested that only micrometastases (and not ITCs) should impact the adjuvant treatment administered, recent data have indicated that patients with ITCs and otherwise low risk factors may experience poorer progression-free survival, although their overall survival remains unaffected [20].

These findings further emphasize the necessity of detecting SLNs in all patients, ensuring bilateral detection, and assessing paraaortic lymph nodes for potential skip metastases, as even patients with ITCs may ultimately benefit from some form of adjuvant treatment. Although a recent retrospective study (SENECA) led to promising results that suggest that the positivity of SLNs may be predicted through the molecular genetic analysis of the tumor, a subsequent prospective study (PROME) did not corroborate these findings [21,22]. Consequently, the SLN biopsy method remains a cornerstone in assessing the spread of tumors to lymph nodes in the management of endometrial cancer patients. Further research conducted in large number of patients, especially or at least in obese patients, is required.

## 5. Conclusions

Our study confirmed that the combination of two tracers (technetium-99 [Tc] and indocyanine green [ICG]) yielded the highest unilateral and bilateral detection rates and the lowest incidence of “empty pockets”; notably, the outcome was also excellent in obese patients. The use of a combination of tracers may reduce the need for site-specific lymphadenectomy and enhance the detection of sentinel lymph nodes (SLNs) for ultrastaging purposes. A discrepancy was observed in 3% of hemipelvices, where the sentinel lymph nodes (SLNs) identified by Tc and ICG were different, potentially leading to a risk of false negative staging.

## Figures and Tables

**Table 1:** General data, histopathologic characteristics, and FIGO stage of the disease of the included patients. BMI—body mass index; SD—standard deviation; LVI—lymphovascular invasion.

Age (Average)	65.9 (SD = 10.29)
BMI (average)	30.7 (SD = 5.31)
Number of pregnancies (average)	2.2 (SD = 1.44)
Number of deliveries (average)	1.6 (SD = 0.99)
Postmenopausal status (yes)	72/86 (83.7%)
FIGO (2009) stage of disease (final surgical stage)	IA 35/86 (40.7%) IB 24/86 (27.9%) II 9/86 (10.5%) IIIA 0/86 (0%) IIIB 0/86 (0%) IIIC1 15/86 (17.4%) IIIC2 3/86 (3.5%)
Endometrioid tumor type	67/86 (77.9%)
tumor differentiation (G2/3 disease)	60/86 (69.8%)
Absence of LVI	55/86 (64.0.0%)
Deep myometrial invasion	48/86 (55.8%)
Involvement of serosa	3/86 (3.5%)
Extrauterine disease	10/86 (11.6%)

**Table 2:** Detection rate and sensitivity of SLN biopsy for lymph node staging and the incidence of “empty pockets” according to the body mass index (BMI) of the patients. Tc—radioactive tracer; ICG—indocyanine green.

	BMI < 30 (39 Patients)	BMI ≥ 30 (47 Patients)	*p*-Value
Detection rate—TC	65.4% (53.8–75.8)	77.7% (67.9–85.6)	*p* = 0.013
Detection rate—ICG	88.5% (79.2–94.6)	90.4% (82.6–95.5)	*p* = 0.083
Detection rate—both	88.5% (79.2–94.6)	97.9% (92.5–99.7)	*p* = 0.013
Sensitivity of SLN—Tc	100% (92.5–100)	97.1% (89.8–99.6)	0.236
Sensitivity of SLN—ICG	100% (94.2–100)	97.4% (91.0–99.7)	0.204
Sensitivity of SLN—both tracers	100% (94.2–100)	97.7% (91.8–99.7)	0.224
Empty pockets—Tc alone	0/0	1/7(14.3%) [0.4%, 57.9%]	1
Empty pockets—ICG alone	0/18 [0%, 18.5%]	4/19 (21.1%) [6.1%, 45.6%]	0.105
Empty pockets—Tc and ICG	2/51 (3.9%) [0.5%, 13.5%]	1/60 (1.7%) [0%, 8.9%]	0.593

## Data Availability

Data are contained within the article.
